# Proteasome: Role in T Cell Function Regulation

**DOI:** 10.7150/ijbs.125134

**Published:** 2025-11-05

**Authors:** Dongyang Tang, Xiaoran Wu, Josh Haipeng Lei, Yunfeng Qiao, Chu-Xia Deng

**Affiliations:** 1 Cancer Center, Faculty of Health Sciences, University of Macau, Macau SAR, China.; 2 Center for Precision Medicine Research and Training, Faculty of Health Sciences, University of Macau, Macau SAR, China.; 3 MoE Frontiers Science Center for Precision Oncology, University of Macau, Macau SAR, China.

**Keywords:** proteasome, proteasome inhibitors, autoimmune diseases, anti-tumor immunity

## Abstract

The proteasome plays a pivotal role in proteostasis and is deeply involved in various cellular processes. Currently, three proteasome inhibitors have been used for clinical therapies of liquid cancers with favorable efficacy, however they fail to achieve ideal efficiency in clinical trials for solid cancers without a clear clue. Recent studies have unveiled that beyond its canonical role in ubiquitin-mediated protein degradation, the proteasome also elicits a multifaceted influence on T cell fate, steering it through antigen processing, metabolic reprogramming, and the prevention of exhaustion. The proteasome inhibitors may affect tumor progression through their critical role in modulating T cell-mediated antitumor immunity, an understanding of which may solve the mystery underlying the poor efficacy of the proteasome inhibitors for solid cancers and unlock novel strategies for precision immunotherapy. This review will summarize the current knowledge of how proteasome activity weaves its threads through thymic selection, T cell aging, activation, differentiation, and immune evasion. Moreover, we will explore how cutting-edge technologies-CRISPR editing, single-cell proteomics, and AI-driven drug design can expand the application of the proteasome inhibitors in the treatment of cancer and autoimmune diseases.

## 1. Introduction

In 2004, the Nobel Prize in Chemistry was awarded to Prof. Aaron Ciechanover, Prof. Avram Hershko, and Prof. Irwin Rose for their groundbreaking discovery of ubiquitin-mediated protein degradation, a process primarily mediated by the proteasome. Recent advancements have unveiled that the proteasome's degradation capabilities extend beyond ubiquitin-tagged proteins, encompassing even those devoid of ubiquitin labels 1. This degradation of non-ubiquitinated proteins is orchestrated by Midnolin, which engages in direct interactions with proteasome subunits 1, 2. The proteasome, with its unique and indispensable functions, plays a pivotal role in proteostasis and is deeply involved in key cellular processes, including cell cycle regulation, DNA damage repair, cell death regulation, drug resistance, inflammatory responses, immune responses, and more 3-9.

**Figure 1:**
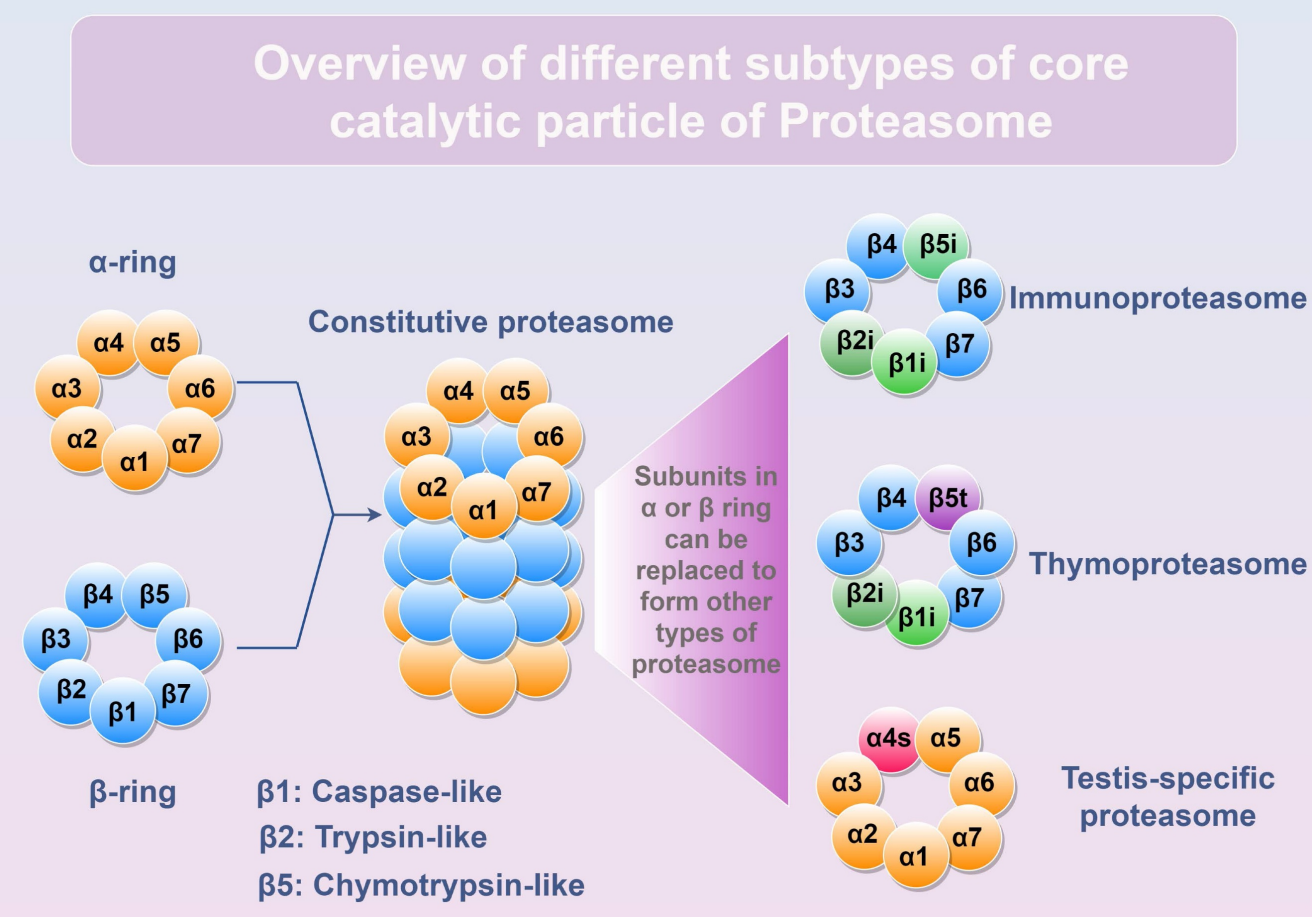
** Different subtypes of core catalytic particle of Proteasome.** The α1-7 or β1-7 subunits form α-ring or β-ring, respectively. The core catalytic particle is assembled by two α rings and two β rings. Subunits inside α or β ring can be replaced to generate different types of proteasomes. β1, β2, and β5 subunits are the proteolytic subunits inside the constitutive proteasome. In immune cells, β1i, β2i, and β5i can replace β1, β2, and β5 to form immunoproteasome. In thymus, β1i, β2i, and β5t replace β1, β2, and β5 to form thymoproteasome. In testis, α4s subunit can replace α4 to form testis-specific proteasome. Figure was generated at FigDraw.

Proteasome is a huge protein complex that manifests in diverse subtypes (26S, 30S, or Hybrid proteasome) through the interplay of core catalytic particle (CP, including Constitutive proteasome, Immunoproteasome, Thymoproteasome, and Testis-specific proteasome) with various regulatory particles (RP, including PA700, PA28αβ, PA28γ, PA200, and PI31) 10. By binding with different RPs, the assembled proteasome exhibits distinct substrate specificity and degradation rates 11. Within the constitutive 20S CP, there exist 4 heptameric rings arranged in the order of α1-7/β1-7/β1-7/α1-7, with the β ring responsible for degrading cargo delivered to proteasome. Among the seven β-subunits (β1-β7), three-β1/PSMB6, β2/PSMB7, and β5/PSMB5-possess proteolytic capacity (Figure 1). However, in immune cells, these three catalytic subunits are replaced by (β1i/LMP2, β2i/MECL1, and β5i/LMP7) to form immunoproteasome 12. Notably, upon exposure to interferon (IFN)-γ, tumor necrosis factor (TNF)-α or other stimuli, the expression of β1i, β2i, and β5i is induced and incorporated into newly synthesized 20S CP in normal cells, leading to the assembly of the immunoproteasome and the execution of its biological functions (Table 1). The immunoproteasome is more efficient at generating antigen peptides for loading onto major histocompatibility complex class I (MHC-I) than the constitutive proteasome 13, thereby possessing greater potency in activating T cell-mediated immunity.

**Figure 2:**
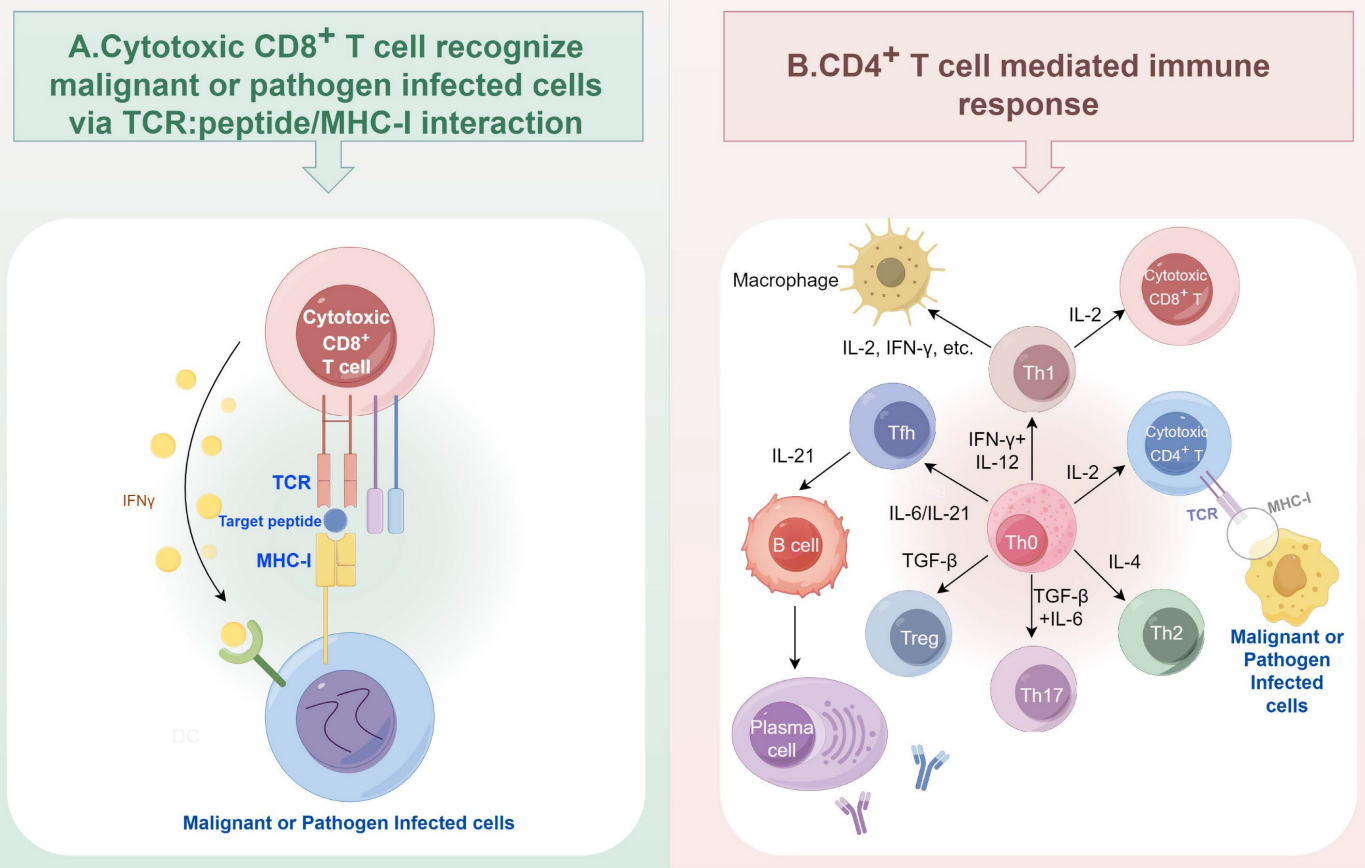
** T cell mediated immune response.** (A) Cytotoxic CD8^+^ T cells eliminate malignant or pathogen infected cells via TCR: peptide/MHC-I interaction. (B) Major subtypes of CD4^+^ T cells and their interaction with macrophages and B cells. Figure was generated at FigDraw.

T cell mediated immune response is a cornerstone of adaptive immunity. Activated CD8^+^ T cells, known as cytotoxic lymphocytes (CTLs), rely on their T cell receptor (TCR) to recognize and eliminate the malignant cells or pathogen- infected cells via recognizing the peptide presented by MHC-I molecules (Figure 2A). CD4^+^ T cells are another major subtype of T cells, which are composed of several major subtypes, including Th1, Th2, Th17, regulatory T (Treg), T follicular helper (Tfh), cytotoxic CD4^+^ T cells, and so on. The different subtypes of CD4^+^ T cells can be differentiated from Th0 cells upon the exposure of different cytokines 21. They mainly secrete cytokines to assist the activation of other immune cells, including macrophages and B cells (Figure 2B). Each subset of CD4^+^ T cells can have both anti-tumor and pro-tumor functions 22. For example, the IFN-γ released by Th1 cells can recruit and enhance the proliferation and cytotoxicity of CD8^+^ T cells 23, while the sustained exposure to this cytokine can lead to tumor growth and metastasis 24. In anti-cancer immunity, defects in antigen processing and presentation represent a key mechanism by which tumor cells escape surveillance by both cytotoxic CD8^+^ T cells and CD4^+^ T helper cells 25-27. During the TCR signal-mediated T cell activation process, proteasome also plays critical roles. Proteasome can be a negative regulator for TCR signal by degrading T cell specific tyrosine kinase p56lck 28, and T cell development essential adaptor protein SLP-76 29 to suppress T cell activation. The proteasome also acts as a positive regulator. Since TCR activation increases the demand for protein turnover, the proteasome is essential for maintaining T cell function 30.

Beyond its well-recognized role in antigen generation to facilitate T cell-mediated immunity, the proteasome also critically regulates T cell survival and differentiation, as highlighted by recent research. Especially, the approval of proteasome inhibitors in clinical practice for cancer treatment marked a milestone, spurring exploration of their immunomodulatory functions. In this review, we summarize current understanding of proteasome-mediated regulation in T cell immunity and evaluate the therapeutic potential of proteasome inhibitors for immune system modulation.

## 2. Functions of proteasome to regulate T cell fitness, development and differentiation

**Figure 3:**
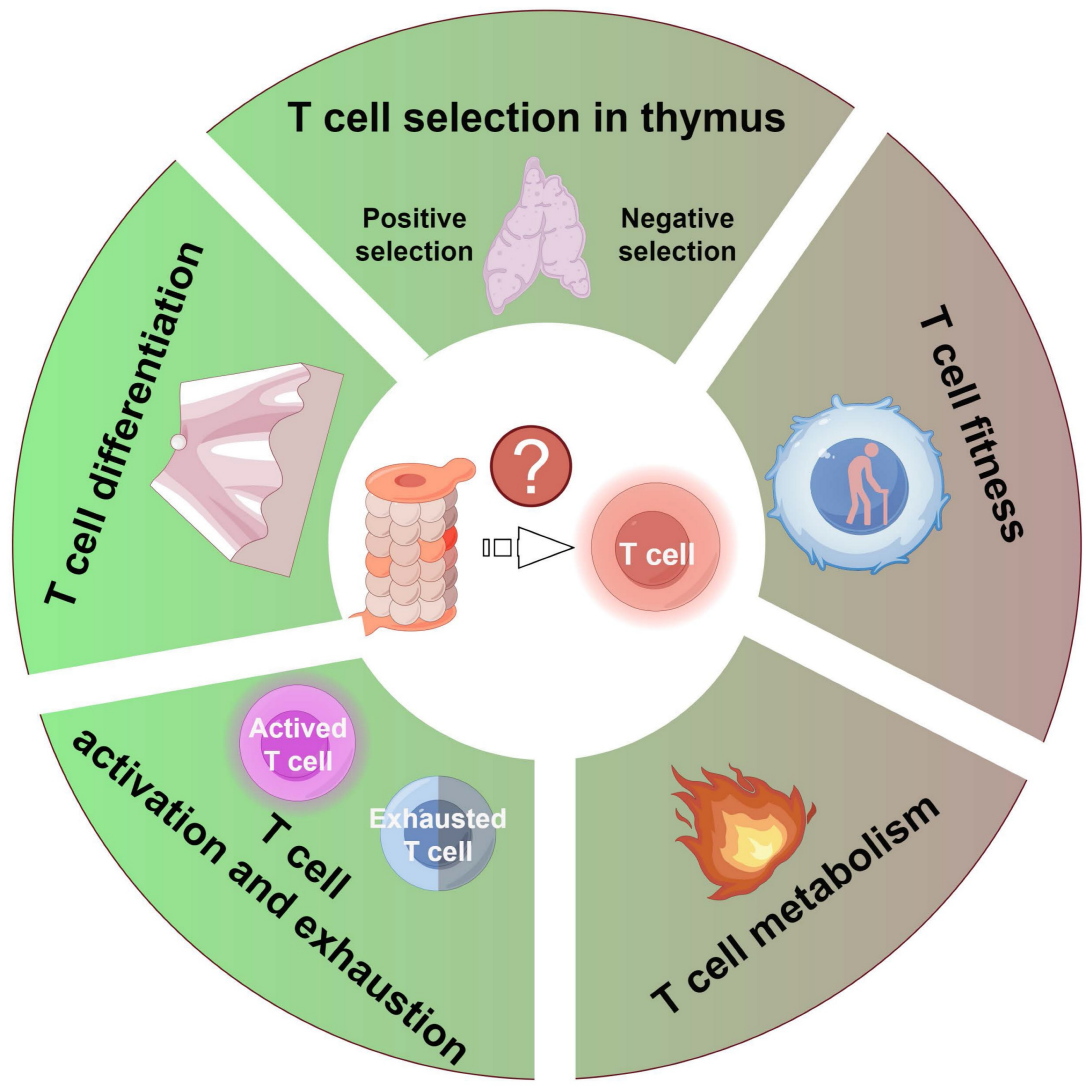
** Proteasome activity exerts a pivotal influence on T cell function by modulating several crucial aspects:** 1) the positive and negative selection of T cells within the thymus; 2) the fitness of T cells due to proteostasis dysfunction; 3) T cell metabolism; 4) the activation and exhaustion processes of T cells; and 5) the differentiation process of T cells. Figure was generated at FigDraw.

Given the proteasome's indispensable role in maintaining cellular homeostasis and regulating diverse signaling pathways, its activity critically governs T cell function at multiple levels. This section discusses how proteasome activity modulates T cell-mediated immune responses (Figure 3).

### 2.1 Proteasome and thymic selection process

In the intricate microcosm of the thymus, T cells undergo both positive and negative selection, a journey that begins in the cortex and traverses into the medulla 31. Within cortical thymic epithelial cells (cTEC), the β5t/PSMB11 subunit together with the β1i/LMP2, β2i/MECL1 forms thymoproteasome 32. Notably, the incorporation of β5t into the thymoproteasome results in a decrease in chymotrypsin-like activity, as compared to proteasomes incorporating β5 or β5i 33. This specialized thymoproteasome generates unique cleavage motifs, which are loaded onto low-affinity TCR ligands, thereby facilitating the specific positive selection of functionally competent CD8^+^ T cells 33, 34. However, aberrant expression of thymoproteasome outside of thymus cause the dysfunction of CD8^+^ T cells homeostasis, accumulation of exhausted or memory CD8^+^ T cells, and autoreactive T cell response 35. In addition, the polymorphisms of PSMB11, especially G49S mutation, have been implicated in influencing T cell selection 36. After navigating the positive selection gauntlet in the cortex, T cells proceed to undergo negative selection in the medulla, a process mediated by medullary thymic epithelial cells (mTECs). These cells express β5i/LMP7, contributing to the formation of the immunoproteasome. Utilizing the lymphocytic choriomeningitis virus (LCMV) infection model, it was found that mice lack of LMP7 cannot generate glycoprotein (GP)_118-125_-specific CTL response 37. Thus, proteasome displays a critical role in T cell development in thymus and the generation of cytotoxic T cell repertoire.

### 2.2 Proteasome and T cell fitness

Dysfunction of proteostasis, a hallmark of aging, has been implicated in the diminished functionality of T cells 38, 39. One pivotal factor contributing to this loss of proteostasis is the gradual decline in proteasome activity with age. In human T cells, the aging dependent proteasome activity loss results in decline of IL-2 receptor expression in response to TNF-α stimulation 40. Moreover, CD4^+^ T cells treated with proteasome inhibitors exhibited a senescence-associated phenotype through accumulating p21 and p27 to induce cell cycle arrest and stabilizing p53 to induce cell apoptosis 30, 41. Of note, the specific knockout of *Rpn13*, a proteasome subunit with ubiquitin binding function, in T cells results in an increased frequency of PD-1^+^/CD44^High^/CD4^+^ T cells upon TCR stimulation, indicating the onset of T cell senescence 30. Collectively, these studies suggested a potent anti-aging role of proteasome in T cells, which is crucial for the combating of age-related decline of T cell function.

In addition, proteasome also acts as a critical but double-edged sword in T cell leukemogenesis, whose effect is determined by which key regulatory proteins it targets for destruction 42, 43. Wang et al. reveal that Hsp90 chaperone can prevent the degradation of oncogenic driver intracellular Notch1 (ICD1) by proteasome and therefore promote cancer progression. Thus, inhibiting Hsp90 re-arms the proteasome to destroy the ICD1 42. Conversely, Jiang et al. show that the Aurora B kinase (AURKB) mediated c-Myc phosphorylation at serine 67 competes with its phosphorylation at threonine 58, which serves as a signal for c-Myc degradation. Ultimately, the AURKB-c-Myc axis promotes T cell leukemogenesis 43.

### 2.3 Proteasome and T cell metabolism

A study conducted by Widjaja et al. revealed that proteasome activity is directly linked to CD8^+^ T lymphocyte metabolism 44. Inhibition of proteasome activity in CD8^+^ T cells results in the stabilization of c-Myc, a transcription factor critical for the controlling of glycolysis 44. This groundbreaking research underscores the proteasome's pivotal role in directly modulating T cell metabolism by regulating the stability of crucial proteins integral to energy production and metabolic pathways.

### 2.4 Proteasome and T cell activation or exhaustion

#### 2.4.1 Proteasome regulating CD4^+^ T cell function

The suppression of proteasome in CD4^+^ T cells results in the loss of activation-associated and functional receptor expression on the cell surface, including CD25, CD28, CD27, CD120b, CD95 and CD134. Additionally, it impairs the production of cytokines such as IFN-γ, TNF-α, IL-4 and IL-5 upon dendritic cell activation. This phenomenon is attributed to the inhibition of nuclear translocation and abundance of nuclear factor of activated T cells (NFAT) c2, ultimately disrupting T cell function 41. As proteasome directly control *de novo* protein synthesis via decreasing intracellular ATP level 45, it is plausible that the inhibition of proteasome activity hinders the translation of these cytokines. Moreover, studies have observed impaired proteasome activity, cell cycle arrest, and cell apoptosis in T cells following the introduction of a β5i specific mutation (G197V) along with *Psmb5* specific knockout 46. Interestingly, another study utilizing an *in vitro* CD4^+^ T cell activation model indicated that inhibition of proteasome activity can reduce the percentage of activation induced cell death through reducing the activity of nuclear factor-κB (NF-κB) 47. Collectively, these studies illuminate the proteasome's multifaceted role in preserving the functional integrity of T cells, underscoring its intricate involvement in regulating various aspects of T cell biology.

#### 2.4.2 Proteasome regulating CD8^+^ T cell function

For CD8^+^ T cells, it was found that Bortezomib treatment of tumor-bearing mice resulted in elevated levels of Notch signal components in lymphoid tissues. This, in turn, upregulated the secretion of IFN-γ and the expression of effector molecules such as perforin, granzyme B, and the T-box transcription factor eomesodermin 48. Importantly, Bortezomib treatment could rescue the dysfunction state of CD8^+^ T cells in tumor microenvironment by enhancing the crosstalk between Notch intracellular domain and NF-κB 48. Moreover, the proteasome plays a crucial role in the degradation of the RNA-binding protein Regnase-1 in T cells. Regnase-1 facilitates the transcription of c-Rel, Ox40, and IL-2 transcripts, thereby acting as a restraint on T cell activation 49.

In addition, it was recently reported that the hyperaction of translation in CD8^+^ T cells leads to increased proteotoxic stress and drives T cell exhaustion 50-52. As a result, pharmacological activation of proteasome was reported able to prevent T cells exhaustion, a status often observed when undergoing chronic infections or combating with cancer, and increase their capacity to fight against cancer 53. The mechanisms behind that are exhausted T cells experience high level of oxidative stress to accumulate oxidized proteins, which will inevitability impair their fitness and function 53. Therefore, the activation of proteasome relieves proteotoxic stress inside T cells when undergoing TCR stimulation and prevents the entry into exhaustion status. Utilizing a high-throughput screening strategy, a novel molecule named compound A was found able to bolster immunoproteasome activity and antigen presentation to increase the anti-tumor efficacy of both allogenic and autologous T cells 54. Collectively, these studies revealed that the proteasome could either activate T cells or induce T cells exhaustion based on different contextual conditions.

### 2.5 Proteasome and T cell differentiation

The proteasome was also found to play a critical role in regulating the differentiation and fate decisions of T cells. The E3 ligase Cul5 was revealed as a critical factor to determine the decision of CD4^+^ T cell differentiated into Treg or Th cells by regulating the IL-4 receptor signaling 55. CD8^+^ T cells undergo asymmetric segregation during their initial division upon encountering microbial infections 56, resulting in daughter cells exhibiting varied proteasome activity 44. Analysis of CD8^+^ T cells derived from *Listeria monocytogenes* infected host showed the daughter cells with low proteasome activity (IL-2Ra^High^/CD62L^Low^) tend to differentiate into terminal effector fate, whereas the daughter cells (IL-2Ra^Low^/CD62L^High^) with high proteasome activity will tend toward a long-lived memory state. Proteasome activation diminishes Myc expression, steering T cell differentiation towards the memory phenotype and disrupting the balance between effector and memory T cell populations 44. Furthermore, the age-related decline in proteasome activity contributes to the differentiation of T cells into effectors rather than memory cells 39.

In the auto-immune or inflammatory disease scenario, the proteasome's activity is also involved. For example, in the experimental Sjögren's syndrome model, LMP7 is observed to be highly expressed within Th17 cells 57. Consequently, inhibiting proteasome activity via Bortezomib suppresses the differentiation of Th17 cells, thereby alleviating the syndrome's manifestations 57. Furthermore, the repression of LMP7 has also been found to mitigate the inflammatory phenotype in the brain associated with LCMV-induced meningitis 58. In the case of Sézary syndrome, it was found that dysfunction of proteasome in T cell led to the accumulation of cytotoxic T-lymphocyte antigen-4 (CTLA-4) and GATA binding protein 3 (GATA3). This accumulation, in turn, facilitates Th2 differentiation and impairs T cell proliferation and responsiveness 59. Furthermore, treating the CD4^+^ T cell mediated experimental autoimmune neuritis (EAN) model with PR-957 to inhibit the immunoproteasome reduced the syndrome of this disease via suppressing the differentiation of Th17 cells 60. Similarly, in a colitis model induced by DSS, it was discovered that proteasome inhibition abolished the differentiation capacity of Th17 cells by impeding the activation of naïve T cells. Notably, this inhibition did not impact the survival of already differentiated Th17 and Treg cells 61. These findings underscore the intricate involvement of proteasome activity in the pathogenesis of autoimmune and inflammatory diseases, highlighting the role of proteasome in maintaining homeostasis.

## 3. Proteasome modulation to shape T cell response

Given the importance of proteasome in T cells as described above, the prospect of harnessing proteasome inhibitors or activators for therapeutic interventions in cancer or T cell-related autoimmune diseases becomes an alluring endeavor. To this end, we have revisited the current repertoire proteasome inhibitors and activators and summarized them in Table 2.

### 3.1 Direct modulation of T cell function by proteasome inhibitors

Given the widespread clinical and preclinical use of proteasome inhibitors, this review focuses on their role in modulating T cell responses. As monotherapy, these inhibitors have been investigated for cancer and autoimmune disease treatment due to their capacity to either augment or suppress T cell activity. In tumor bearing mouse models, Bortezomib treatment did not affect the total number of lymphocytes, and dendritic cells 92. Also, this treatment did not reduce the levels of costimulatory molecules (CD80, CD86, and CD40) in the dendritic cells and its ability to present MHC associated antigen to T cells. Instead, Bortezomib treatment increased the phosphorylation of NF-κB p65 in CD8^+^ T cells, which induce the expression of CD3ζ and IL-2 receptor-α and maintain the section of IFN-γ, suggesting the activation of CD8^+^ T cell function *in vivo*. In the context of adoptive T cell therapy, Bortezomib was combined with adoptive T-cell immunotherapy to treat the* Rag2^-/-^* tumor bearing mice and observed reduced lung metastasis and increased survival rate 92. Notably, two independent studies revealed the co-treatment with proteasome inhibitors (Bortezomib or Carfilzomib) boost the efficiency of anti-B cell maturation antigen (anti-BCMA) Chimeric antigen receptor T (CAR-T) cell in multiple myeloma animal model and human patients via blocking the proteasome mediated degradation of BCMA to stabilize its in cell membrane 93, 94. In melanoma, Bortezomib treatment sensitizes cancer cells to the attack of antigen specific cytotoxic T lymphocytes (CTL). Mechanistically, it was found that Bortezomib treatment induced the accumulation of NOXA to stimulate the release of second mitochondria-derived activator of caspase (SMAC) from mitochondrial upon the attack from effector proteins caspase 8 and granzyme B release from CTL 95. In malignant mesothelioma, Bortezomib treatment induced the generation and recruitment of functional CD4^+^ and CD8^+^ T cells 96. These studies highlight the potential of proteasome inhibitors to enhance antitumor T-cell function in cancer immunotherapy.

### 3.2 Indirect modulation of T cell function by proteasome inhibitors

**Figure 4:**
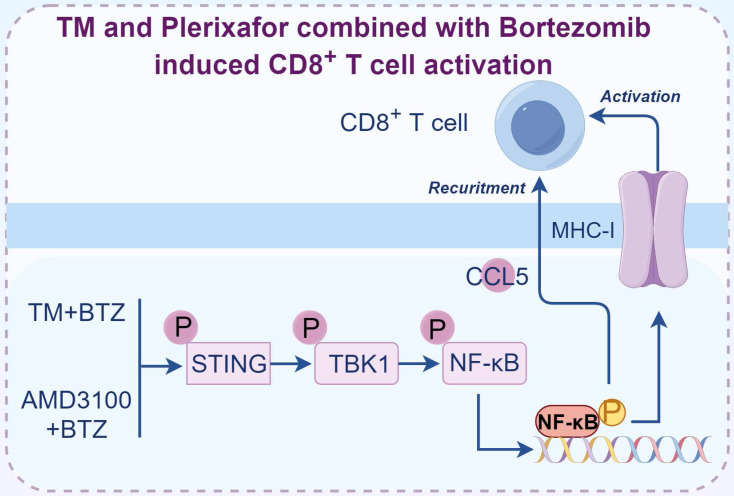
** Representative proteasome inhibitor centered combination therapy for activating T cell mediated immune response.** The combination of TM with bortezomib or plerixafor with bortezomib induced the generation of cytotoxic, antigen-specific CD8^+^ T cells in breast cancer. Figure was generated at FigDraw.

In addition to their direct modulation of T cell function, recent research has illuminated the indirect influence that proteasome activity regulators exert on T cell performance by reshaping the tumor microenvironment. The suboptimal outcomes observed with proteasome inhibitors in the treatment of solid tumors 97 have prompted a shift towards exploring the combination of proteasome inhibitors with other drugs, which have achieved variably enhanced therapeutic effects (Table 3 and Figure 4), and stimulated the activation of the immune system to fight against solid tumors 98-102.

Realizing the combination of proteasome inhibitors with different drugs illicits variable effect, we suspected that the drug pairs used by different studies might not be optimal. To select the drug combinations with higher therapeutic efficacy, we employed a drug combination screening approach to select FDA-approved drugs that can augment the efficacy of Bortezomib 99. To this end, we selected both *BRCA1* wild-type and mutant breast cancer cells to do screening with the aim to identify effective drug pairs. After screening the 115 FDA-approved drugs, we found only two drugs, TM and Plerixafor, were able to significantly enhance the efficacy of Bortezomib in inhibiting the growth of breast cancer cell lines, tumor slices, and organoids. Interestingly, when we applied these drug combinations for breast cancer treatment *in vivo*, the tumor inhibition effects were only observed in immune intact mice but not the immunodeficient mice, highlighting the importance of T cells in mediating the killing effects of drug combinations 99. The killing effects of drug combinations were compromised when utilizing anti-CD8α antibody to deplete CD8^+^ T cells in immune intact mice, suggesting drug combinations treatment induces the generation and expansion of cytotoxic CD8^+^ T cells, which are responsible for the inhibition of tumor growth. To explore the detailed mechanisms that mediate the activation of CD8^+^ T cells, we analyzed the drug combinations treated tumor tissues and found tumor microenvironment was reshaped through CCL5 mediated CD8^+^ T cell recruitment. In the meantime, we showed that the cancer cells displayed high levels of MHC-I upon drug combination treatment mediated by the activation of STING/TBK1/NF-κB pathway 99. Taken together, we provided a strategy that inhibiting proteasome activity in cancer cells promotes the production of MHC-I and CCL5, which facilitates the activation of CD8^+^ T cells.

## 4. Challenges in targeting proteasome for therapeutic purposes

The clinical use of proteasome inhibitors for cancer treatment provided a proof-of-concept foundation for targeting proteasome to treat human diseases. Future efforts are focused on optimizing these strategies for broader clinical use, with a nuanced understanding of their complex immunomodulatory effects. This involves a delicate balancing act: enhancing antitumor immunity while mitigating the disruption of immune homeostasis.

### 4.1 Debates on proteasome inhibition in disease treatment

Debates persist regarding the therapeutic efficacy and immunomodulatory effects of proteasome inhibitors across diseases. In autoimmune contexts, their application remains contentious. For rheumatoid arthritis (RA), proteasome inhibitors suppress proinflammatory cytokine release by inhibiting NF-κB activation, yet their long-term disease control efficacy is incompletely established 112, 113. Another debate is the “bounce-back effect” of applying proteasome inhibitors for disease treatment 114. For example, the specific immunoproteasome inhibitors were initially believed to deplete of pathogenic immune cells but not non-hematopoietic cells, however, it was recently found those drugs did not lead to immune cell death as anticipated 115. Subsequent studies revealed that compensatory upregulation of constitutive proteasomes confers cellular protection against immunoproteasome inhibitor-induced apoptosis 115.

In the context of cancer therapy, proteasome inhibitors paradoxically may suppress antigen presentation and NF-κB signaling-potentially dampening host immunity and limiting synergy with immunotherapies 116. Nevertheless, recent evidence demonstrates that tumor cell-targeted proteasome inhibition triggers antitumor CD8^+^ T cell responses 99-101, 104. This suggests cell type-specific outcomes: proteasome inhibition in malignant versus immune cells exerts divergent effects on CD8^+^ T cell functionality.

### 4.2 Challenges in targeting proteasome for therapeutic purpose

Although proteasome inhibitors have been approved for the treatment of multiple myeloma and mantle T cell lymphoma in clinical practice, their utilization in cancer therapy continues to confront several hurdles. Firstly, the currently used proteasome inhibitors, such as Bortezomib and Carfilzomib, can target both the constitutive and immunoproteasome. The lack of specificity of proteasome inhibitors results in adverse effects, such as peripheral neuropathy due to their impact on normal cells, thereby limiting their therapeutic efficacy 117. Furthermore, when these inhibitors are applied to treat cancer cells, they may inhibit the function of cytotoxic immune cells such as nature killer cells, which also rely on protein turnover to produce cytokines 41. Secondly, both intrinsic and acquired resistance to proteasome inhibitors treatment have been observed. In multiple myeloma, some patients are initially resistant or become resistant to treatment of proteasome inhibitors 118. Various resistance mechanisms were involved, including Wnt signal activation, ABCB1 overexpression, and the “bounce-back effect” caused by proteasome inhibitor treatment 114, 119, 120. Thirdly, the three approved proteasome inhibitors in clinical only demonstrate treatment efficacy in liquid tumors but not in solid tumors. This is partly due to insufficient delivery of proteasome inhibitors to solid tumor sites, resulting from their poor penetration and limited tumor blood supply, which compromises efficacy 97. Therefore, identifying novel proteasome inhibitors and exploring proteasome inhibitor-centered drug combination therapies effective against solid tumors is essential. For example, the combination of TM+Bortezomib or Plerixafor+Bortezomib (Figure 4), which significantly inhibit tumor growth in immunocompetent mouse models 99, warrant clinical evaluation to assess their efficacy in human patients. Last but not least, the ethical considerations of proteasome inhibitors for disease treatment, especially the off-label usage of proteasome inhibitors and how to select the appropriate patients for treatment, still require further discussion.

### 4.3 New strategies to deal with the current challenges faced by proteasome inhibitors centered therapy

To address these aforementioned challenges, extensive research has been conducted to explore and develop novel strategies. Regarding the first challenge, increased efforts have been directed towards the discovery of subunit- or subtype-specific proteasome inhibitors, aiming to mitigate side effects while maximizing therapeutic efficacy 121-123. Given that current proteasome inhibitors primarily target the β5/PSMB5 subunit, researchers have developed a β2/PSMB7-specific inhibitor, which has demonstrated the ability to sensitize cancer cells to β5/PSMB5 inhibition 121, 122. To avoid the cross effects of proteasome modulators on different types of cells, it is necessary to develop cell-type specific delivery systems to targeted delivery of reagents to induce death in tumor cells and inhibitory immune cells, while preserving and enhancing the function of tumor killing immune cells. Given that proteasome plays distinct functions in the activation to exhaustion process of T cells 47, 50, 51, the function of T cell should be assessed at first before the selection of proteasome inhibitors or activators for disease treatment to achieve optimal treatment outcomes. To counteract ABCB1 overexpression mediated drug resistance, proteasome inhibitors were combined with ABCB1 inhibitors (Verapamil, Nelfinavir, and Lopinavir) to block the efflux of proteasome inhibitors to the extracellular space, which will definitely increase the concentration of proteasome inhibitors inside the cells to eliminate the cancer cells 119, 120. Addressing the “bounce-back effect” where cancer cells develop resistance to proteasome inhibitors, researchers have identified promising combinations. Specifically, the integration of Cisplatin 124, N-glycanase 1 (NGLY1) inhibitors 125, and bromodomain extra-terminal (BET) inhibitors 126 has shown the capacity to suppress the transcriptional activity of Nuclear Factor, Erythroid 2 Like 1 (NFE2L1, Nrf1). This suppression completely reverses the resistance effect and elicits robust anti-tumor responses from proteasome inhibitors. In response to the insufficient accumulation of proteasome inhibitors in solid tumors, the development of innovative drug delivery strategies or novel inhibitors with superior tumor targeting capabilities is imperative 127, 128. To deal with patient selection challenge, PTEN mutation has emerged as a biomarker predictive of cholangiocarcinoma sensitivity to proteasome inhibitors. Consequently, clinical trials using proteasome inhibitors for cholangiocarcinoma treatment have yielded encouraging results 129, 130. In our recent study, we also proposed that cancer patients with low *STAT3/PRAKK1* or *STAT3/PRAKK2* ratio tend to have better response to Bortezomib treatment compared with those with high ratio. This suggests that measuring and calculating the gene ratio of these two genes may serve as novel predictive biomarkers for Bortezomib treatment outcomes in cancer patients 99. Despite the significant challenges, identifying specific biomarkers for predicting proteasome inhibitor treatment outcomes remains a crucial endeavor. At last, the tumor immunomicroenvironment reshaping capability of proteasome modulators lays a foundation to combine proteasome-centered therapies with immune checkpoint inhibitors, CAR-T cell therapy, and HDAC inhibitors. In conclusion, the therapeutic landscape of proteasome modulation is rapidly advancing from simple inhibition to sophisticated immunomodulation. By leveraging the above-described approaches, we can unlock the full potential of proteasome modulators to treat cancer and autoimmune diseases with greater efficacy and precision.

## 5. Conclusion

In this review, we have systematically delineated the current understanding of proteasome's activity in orchestrating T cell function. We have delved into the role of proteasome inhibitors in modulating the anti-tumor effects of T cells, explored combination strategies to augment the therapeutic efficacy of proteasome inhibitors in cancer treatment, and examined their impact on T cell activation. Given the proteasome's indispensable and unique function within cells, as well as its pivotal role in the pathogenesis of numerous human diseases, modulating proteasome activity stands out as an alluring therapeutic strategy, promising to pave the way for personalized medicine. The proteasome plays a critical role in modulating T cell function, and its inhibition holds promise as a strategy to forestall T cell dysfunction, thereby overcoming a formidable hurdle in cancer immunotherapy. As research in this domain progresses, the immunoproteasome is poised to remain a key target for regulating T cell function and treating a spectrum of diseases, including cancer, autoimmune disorders, and neurological conditions 131. The development of more specific immunoproteasome inhibitors and an in-depth understanding of their long-term immunological effects will be indispensable in advancing this field. As our insights into the proteasome's mediation of immune crosstalk deepen, targeting these intricate interactions emerges as a viable therapeutic approach for personalized immunotherapies. Personalized proteasome-targeted therapies, meticulously tailored to individual genetic profiles, immune statuses, and disease characteristics, hold the potential to usher in more efficacy and less toxic treatment modalities in the future. The continued exploration of proteasome research in immunology is expected to broaden our understanding of immune regulation and disease pathogenesis, heralding new horizons in therapeutic interventions.

### 5.1 Future perspectives on proteasome and T cell mediated immunity

#### 5.1.1 Emerging technology to study the function of proteasome function

To gain a deeper understanding of the diverse roles of the proteasome in various cell populations, the adoption of innovative technologies is imperative. Firstly, CRISPR-Cas technology, with its conventional gene knockout/knock-in capabilities and cutting-edge base editing methods, allows for precise manipulation of genes associated with the proteasome system in T cells. This precision editing accelerates our comprehension of proteasome activity or specific gene mutations within the proteasome influence T cell function 132-135. Secondly, the Mass spectrometry-based proteomics approach offers a means to elucidate the dynamic proteome of the proteasome complex under varying conditions. This could provide novel insights into the mechanisms regulating proteasome function 136. Thirdly, the emerging of activity-based probes facilitates the identification of the spatial localization of the proteasome within cells and tissues 137. Furthermore, high-throughput functional single-cell proteomics emerges as a potent tool to dissect cellular heterogeneity and study proteasome activity across all cells within the tumor microenvironment 138-140. The combination of activity-based probes and single-cell proteomics enables researchers to dissect variations in proteasome activity among different immune cell subsets and elucidate its role in anti-tumor immunity. Building upon these technologies, increasing research focus has been directed towards understanding the complexity of the tumor microenvironment and how specific cell types influence the function of other cell types, particularly the interplay among various cell types. As proteasome modulators treatment could potentially affect the function of different cells and thus their immune outcomes, it is imperative to employ novel *ex vivo* co-culture systems such as 3D-tumor slice culture 141, 142, air-liquid culture 143, and patients derived organoid culture systems 144. These systems allow for the study of the fate of each cell type when exposed to these drugs and how T cell function is affected in complex scenarios. Finally, the emergence of artificial intelligence and machine learning methods holds promise for designing more specific proteasome inhibitors. Future inhibitors could target only the constitutive proteasome while sparing the immunoproteasome, and these methods could predict the interplay between proteasome function and T cell response using current multi-omics data. These cutting-edge technologies and advancements will undoubtedly aid in deciphering the intricate interactions between proteasome function and T cell response.

#### 5.1.2 Future directions in proteasome and T cell research

Future research in proteasome and T cell research is likely to focus on several key areas. A recent study utilizes mRNA vaccines to express fusion peptide of antigen and a proteasome-targeting peptide that can be processed by proteasome to present antigen on the cell surface. By utilizing this mRNA vaccine, they observed very strong CD8^+^ T cell mediated immune response 145. In the context of CAR-T cell therapies for cancer, there is a need to further explore the optimal treatment strategy including treatment dosage, frequency, and treatment route of proteasome inhibitors when combined with the BCMA targeted CAR-T cells 93, 94, 146. This also involves extending these findings and treatment strategy to other CAR-T cells in addition to the BCMA targeted CAR-T cells. For the complex function of proteasome in T cell development, differentiation, survival, activation, and exhaustion, more efforts are needed to clearly define the impact of proteasome activity on different types and their developmental stages of T cells and also the optimal strategy for intervention. Furthermore, a deep understanding of the complex dynamics of proteasome in tumor microenvironment and the role of the proteasome in immune activation and evasion will be crucial for the development of these new therapeutic approaches.

## Figures and Tables

**Table 1:** Stimuli that can induce the expression of immunoproteasome subunit

Stimuli	Impact	Ref.
IFN-γ	Induces the transcription and translation of immunoproteasome subunits, PA28αβ, and PA28γ	14
TNF-α	Induces the transcription and translation of immunoproteasome subunits. Often synergy with IFN-γ.	15
Retinoic acid	Induces the transcription and translation of immunoproteasome subunits.	16
Nitric oxide	Induces the expression of immunoproteasome subunits (β1i and β5i).	17
Toll-like receptor agonists	Induces the expression of immunoproteasome subunits (β5i).	18
Type I interferons	Induces the transcription and translation of immunoproteasome subunits	19
Mammalian target of rapamycin (mTOR) signaling	Promotes immunoproteasome formation via PRAS40 phosphorylation	20

**Table 2:** List of known proteasome inhibitors and activators

Category	Name	Type	Status	Ref.
Inhibitor	Bortezomib	Dipeptide boronic acid/Reversible inhibitor	Approved to treat multiple myeloma and mantle cell lymphoma	62
	Carfilzomib	Epoxyketone-peptide/Irreversible inhibitor	Approved to treat multiple myeloma and mantle cell lymphoma	63
	Ixazomib	Dipeptide boronic acid/Reversible inhibitor	Approved to treat multiple myeloma and mantle cell lymphoma	64
	MG132	Peptide derivates/Reversible inhibitor	Preclinical	65
	MG262	Peptide derivates/Reversible inhibitor	Preclinical	66
	Marizomib	β-lactone-γ-lactam /Irreversible inhibitor	Phase III clinical trial to treat glioblastoma	67, 68
	Oprozomib	Tripeptide epoxyketone/Irreversible inhibitor	Phase Ib/II clinical trial to treat multiple myeloma	69, 70
	Delanzomib	Boronic acid peptide/Reversible inhibitor	Phase I/II clinical trial to treat multiple myeloma	71, 72
	Epoxomicin	Epoxyketone/Irreversible inhibitor	Preclinical	73
	Lactacystin	Cyclic α,α-disubstituted amino acid including a γ-lactam-β-lactone/Irreversible inhibitor	Preclinical	74
	Disulfiram/Cu	Likely targets protein cysteine not at the binding site	Approved to treat chronic alcoholism	75
	Epigallocatechin-3-gallate	Polyphenol/Irreversible inhibitor	Preclinical	76
	Beta-hydroxy beta-methylbutyrate	Leucine metabolite with unidentified mechanism to inhibit proteasome activity	Preclinical	77
	PR-957	Epoxyketone-peptide/ Irreversible immunoproteasome inhibitor	Preclinical	60
	PI31	Endogenous 20S proteasome binding protein	-	78
Activator	PA28α/β	Endogenous RP	-	10
	PA28γ	Endogenous RP	-	10
	PA200	Endogenous RP	-	10
	cAMP	Activate PKA to phosphorylate Rpn6/PSMD11	-	79
	cGMP	Activate PKG to increase phosphorylation on proteasome subunits	-	80
	ZFAND5/ZNF216	Interact with 19S proteasome to induce conformation change	-	81, 82
	Ursolic acid	Triterpenoid	Preclinical	83
	Betulinic acid	Triterpenoid	Preclinical	84
	Oleuropein	Triterpenoid	Preclinical	85
	MK-886	Indole-based compound	Preclinical	86
	TCH-155	Imidazolines	Preclinical	87
	Low concentration (0.04-0.08%) SDS	Synthetic organosulfate salt/Likely via inducing gate opening of the proteasome	Preclinical	88
	Chlorpromazine	Phenothiazines	Approved to treat mental health condition	89
	Pyrazolones	Five-membered lactam ring	Preclinical	90
	Fluspirilene	Allosteric modulate proteasome active site	Approved to treat schizophrenia	91

**Table 3:** Effective proteasome inhibitors centered drug combinations to treat solid tumors

Proteasome inhibitor	Additional drug	Tumor type	Treatment outcome	Ref.
Bortezomib	TM	Breast cancer	Inhibit breast cancer (4T1, EMT6, HP10069, and Py8119) growth in immunocompetent mice through activating CD8^+^ T cell	99
	Plerixafor	Breast cancer		
	Cisplatin	Breast cancer	Inhibit MDA-MB-231 tumor growth in nude mice relied on their direct killing effects on tumor cells (Phase I clinical trial)	8
	Nedaplatin	Breast cancer	Inhibit MCF7 tumor growth in zebrafish xenografts	103
	PD0166285	Liver Cancer	Induction of pyroptosis to inhibit the growth of liver cancer in immunocompetent mice	104
	Vorinostat	Cervical cancer	Induce the generation of anti-specific CD8^+^ T to inhibit tumor growth in immunocompetent mice	101
	Cisplatin	Lung cancer	Synergistically inhibit lung cancer cell growth *in vitro*	105
	Gefitinib	Lung cancer		
	Gemcitabine	Lung cancer		
	Vinorelbine	Lung cancer		
	Paclitaxel, carboplatin, and radiation therapy	Lung cancer	Lung cancer patients treated with those combinations showed increased overall survival (Phase I/II clinical trial)	106
Carfilzomib	Lopinavir	Renal cell carcinoma	Synergistically inhibit renal cell carcinoma cell growth *in vitro* though induction unfolded protein response	107
	Nelfinavir	Renal cell carcinoma		
	HRS-4642	*KRAS G12D*-mutant cancer	Synergistically inhibit the growth of pancreatic cancer, colorectal cancer, and non-small lung cancer through Notch4 inhibition and interferon alpha response activation (Phase I clinical trial)	100
	Irinotecan	Small cell lung cancer	Objective clinical response was 19% (Phase Ib clinical trial)	108
	ONX 0912	Head and neck cancer	Induce cell death via suppression of Mcl-1 or autophagy	109
	CUDC-101	Thyroid cancer	Induce cell death via induction of p21 expression	110
Ixazomib	Dinaciclib	Hepatocellular carcinoma	Synergistically inhibit the growth of human patient derived organoids and xenografts models	111
